# Molecular recognition of AT-DNA sequences by the induced CD pattern of dibenzotetraaza[14]annulene (DBTAA)–adenine derivatives

**DOI:** 10.3762/bjoc.10.225

**Published:** 2014-09-12

**Authors:** Marijana Radić Stojković, Marko Škugor, Łukasz Dudek, Jarosław Grolik, Julita Eilmes, Ivo Piantanida

**Affiliations:** 1 Laboratory for Study of Interactions of Biomacromolecules, Division of Organic Chemistry and Biochemistry, Ruđer Bošković Institute, Bijenička cesta 54, PO Box 180, HR-10002 Zagreb, Croatia; 2 Department of Chemistry, Jagiellonian University, Ingardena 3, 30-060 Kraków, Poland

**Keywords:** AT-DNA recognition, circular dichroism, DBTAA-adenine conjugate, ds-DNA/RNA, minor groove binding, nucleic acids

## Abstract

An investigation of the interactions of two novel and several known DBTAA–adenine conjugates with double-stranded DNA and RNA has revealed the DNA/RNA groove as the dominant binding site, which is in contrast to the majority of previously studied DBTAA analogues (DNA/RNA intercalators). Only DBTAA–propyladenine conjugates revealed the molecular recognition of AT-DNA by an ICD band pattern > 300 nm, whereas significant ICD bands did not appear for other ds-DNA/RNA. A structure–activity relation for the studied series of compounds showed that the essential structural features for the ICD recognition are a) the presence of DNA-binding appendages (adenine side chain and positively charged side chain) on both DBTAA side chains, and b) the presence of a short propyl linker, which does not support intramolecular aromatic stacking between DBTAA and adenine. The observed AT-DNA-ICD pattern differs from previously reported ss-DNA (poly dT) ICD recognition by a strong negative ICD band at 350 nm, which allows for the dynamic differentiation between ss-DNA (poly dT) and coupled ds-AT-DNA.

## Introduction

The majority of natural and artificial applications involving small molecule-DNA/RNA recognition depend on several non-covalent binding modes. Typical examples are double-stranded (ds) DNA/RNA intercalation, minor or major groove binding, and external electrostatic binding [1]. However, non-covalent interactions involving small molecules (*M*_w_ < 600) can only rely on a small number of interacting groups, while the steric parameters of DNA/RNA binding sites are also quite limited. Nevertheless, the dynamic nature of nucleic acids, combined with structural differences between various DNA/RNA sequences, offer numerous highly interesting targets. Within the last two decades, the design of small binding molecules has mostly relied on the three-dimensional recognition of various targeted DNA/RNA sites [2–3], frequently relying upon new knowledge gained from supramolecular chemistry [4].

Among the large number of DNA/RNA sequences of biochemical interest, long homogeneous AT tracts have attracted significant attention as small molecule targets. An illustrative natural example can be found in proteins which use selective binding interactions of an arginine-rich side arm inside the AT sequence minor groove to broaden the related DNA major groove, within which the protein biological action takes place [5]. However, the majority of known small molecules are not able to distinguish AT tracts by length and composition. This has been demonstrated, for instance, by the inability to distinguish between longer tracts (more than 20–30 base pairs) of homo and alternating distribution of AT base pairs.

**Scheme 1:**
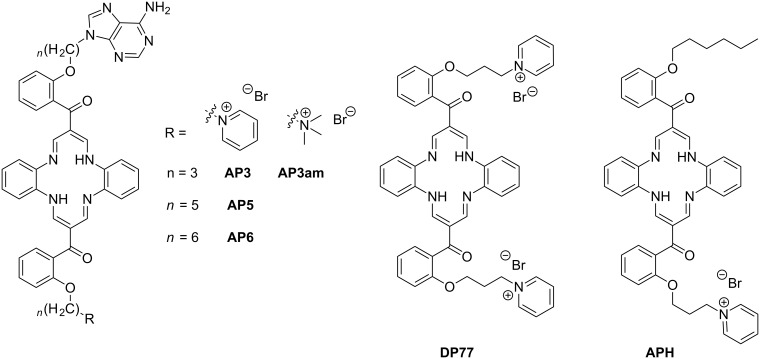
Studied DBTAA–adenine conjugates (**AP3**, **AP3am**, **AP5**, **AP6**) [11], and the reference compounds lacking adenine (**APH** [11], **DP77** [12–13]).

Our recent studies revealed a novel class of cyanine dyes characterized by bulky phosphonium substituents as intriguing AT binders, which showed very rare kinetic differentiation between alternating- and homo-AT-DNA sequences [6]. Moreover, these dyes efficiently entered cells and were shown to be non-toxic, mitochondria-specific fluorescent markers [7]. Previous studies also revealed the advantage of nucleobase incorporation in small molecule structures for the recognition of complementary nucleotides/polynucleotides [8–10]. Recent studies of the novel DBTAA-adenine conjugates **AP3** and **AP6** (Scheme 1) showed a highly selective binding of only **AP3** to poly dT among all other ss-DNA/RNA, as characterized by the induction of a specific CD band response [11]. Observed oligo dT specificity provoked the intriguing question of whether such a specificity would be observed in double-stranded AT-DNA sequences. The experimental design to investigate this question was supported by the aforementioned importance of AT tracts and the selectivity of cyanine dyes. On this basis, we were inspired to prepare the novel binders **AP5** with a DBTAA–adenine linker length between **AP3** (oligo dT specific) and **AP6** (oligo-dT inert) and **AP3am**, in which pyridinium is exchanged by a permethylated amine, with the aim of determining the importance of aromatic stacking interactions. The results were compared with the reference **APH** (lacking adenine) and previously studied **DP77** [12] (Scheme 1), which, having pyridinium instead of adenine, can also be regarded as a reference structure. Double-stranded DNA/RNA targets chosen for this study are long (<100 base pairs) synthetic polynucleotides poly dG–poly dC, poly dA–poly dT, poly dAdT–poly dAdT and poly rA–poly rU, each associated with specific structural properties of the minor/major groove as the anticipated binding site (**APH**, **AP3**, **AP6** didn’t intercalate into ct-DNA [11]). Namely, parameters such as the groove width and depth, steric obstructions like the amino groups of guanine, H-bonding patterns, as well as polynucleotide charge density and the curvature of the ds-helix backbone all differ significantly across the double stranded examples mentioned above (Table S1, Supporting Information File 1). The choice of long polynucleotides ensures that significant binding of the DBTAA moiety at the ends of the double strands (“capping”) can be neglected and that our investigations indeed sense the differences of the secondary structure (from minor/major groove) of the studied DNA and RNA sequences.

## Results and Discussion

### Synthesis

**Scheme 2:**
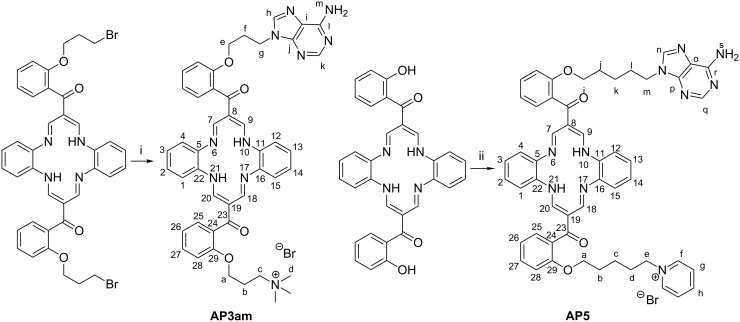
Preparations of the products **AP3am** and **AP5**. Starting compounds were synthesized according to previously reported procedures [13–14]. i: (1) adenine, NaH, DMF, (2) trimethylamine, DMF; ii: (1) 9-(5-bromopentyl)adenine [15], potassium carbonate, DMF, (2) 1,5-dibromopentane, potassium carbonate, DMF, (3) pyridine.

The synthetic routes to the new adenine–DBTAA conjugates **AP3am** and **AP5** are summarized in Scheme 2, the details of which are given in the Experimental section.

### Spectroscopic characterisation of DBTAA derivatives in aqueous medium

Novel compounds **AP3am** and **AP5** were moderately soluble in aqueous solutions, that is, up to *c*_AP3am_ = 1 × 10^−3^ mol dm^−3^ and *c*_AP5_ = 1 × 10^−4^ mol dm^−3^, respectively. Buffered aqueous solutions of the studied compounds were stable for several months and their absorbances were proportional to their concentrations up to *c* = 3 × 10^−5^ mol dm^−3^. Changes of the UV–vis spectra in response to temperature increases of up to 95 °C were negligible, and the reproducibility of the UV–vis spectra upon cooling back to 25 °C was excellent. The UV–vis spectra of new compounds (**AP3am**, **AP5**) were similar to previously studied analogues [11] (Supporting Information File 1).

#### Study of interactions of DBTAA derivatives with ds-DNA/RNA in aqueous medium

Previous studies [11] revealed intriguing recognition of single stranded DNA dT sequences by **AP3** (but not **AP6**, **APH**), whereby intercalation of **AP3**, **AP6**, **APH** in double stranded ct-DNA was excluded. Here, we studied in more detail the interactions of **AP3**, **AP6**, **APH** as well as the novel **AP3am** and **AP5** with a series of synthetic double-stranded polynucleotides.

#### Thermal denaturation experiments

Non-covalent binding of ligands to ds-DNA/RNA usually induces stabilization of the ds-helix against thermal denaturation resulting in the increase of DNA/RNA-*T*_m_. In particular, thermal stabilization is characteristic for the intercalative binding mode due to the strong aromatic stacking interactions between studied condensed aromatic molecule and adjacent base pairs [1,16].

None of the **AP** compounds showed any stabilization effect on any of studied ds-polynucleotides, whereas the previously studied **DP77** analogue [12] showed a significant stabilization effect. The **DP77** analogue essentially differing from the **AP** series by the presence of two positive charges, one each on side arm (Table 1).

**Table 1:** The Δ*T*_m_^a^ values (°C) of studied ds-polynucleotides upon addition of **AP3**, **AP3am AP5, AP6** and **APH** at pH 7.0 (sodium cacodylate buffer, *I* = 0.05 mol dm^−3^), ratio *r* = 0.3^b^.

	**AP3**	**AP3am**	**AP5**	**AP6**	**APH**	**DP77** [12]

ct-DNA (*T*_m_ = 79.5 °C)	0	0	0	0	0	12.8
poly A - poly U (*T*_m_ = 52.7 °C)	0.5	0	0	0	0	28.1
poly dA–poly dT (*T*_m_ = 62.7 °C)	0.7	0	0	0	0	12.2
poly dAdT–poly dAdT (*T*_m_ = 56.0 °C)	<3	<2	–	0	0	–

^a^Error in Δ*T*_m_ : ±0.5 °C; ^b^*r* = [compound]/[polynucleotide].

#### UV–vis titrations and affinity determination

**Figure 1:**
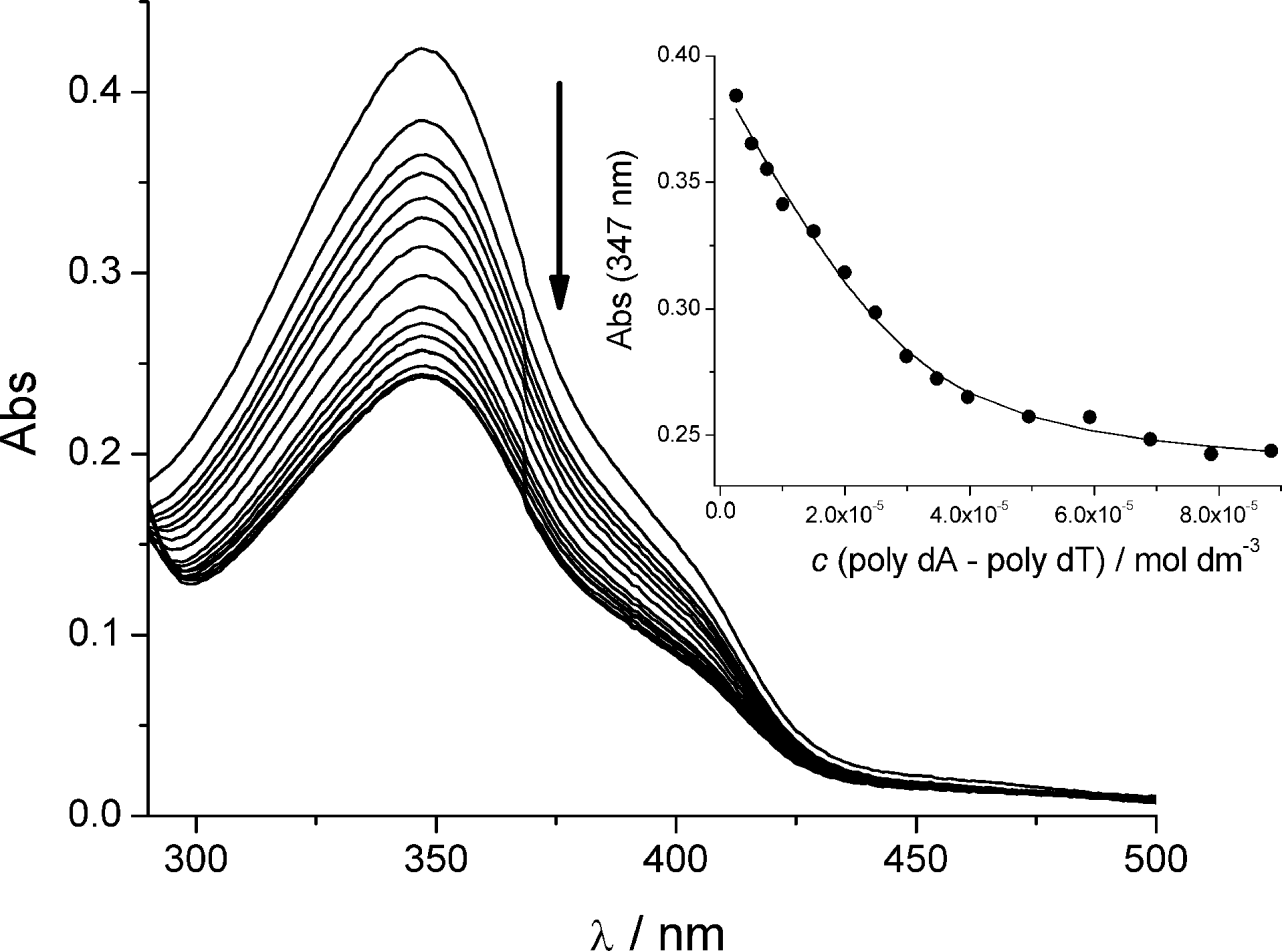
Changes in the UV–vis spectrum of **AP3am** (*c* = 1.0 × 10^−5^ mol dm^−3^) upon titration with poly dA–poly dT; Inset: dependence of the **AP3am** absorbance at λ_max_ = 347 nm on *c* (poly dA–poly dT), pH 7.0, sodium cacodylate buffer, *I* = 0.05 mol dm^−3^.

Titration of the **AP** series with any studied polynucleotide resulted in a pronounced decrease of the UV–vis absorbance of the DBTAA chromophore at >300 nm (Figure 1, Table 2). However, no measurable shifts of the UV–vis absorption maxima for any of the **AP** series were observed. In contrast, the previously studied analogue **DP77** exhibited strong bathochromic shifts of (Δλ_346 nm_ = 4–13 nm; pyridinium instead of adenine) [12]. The observation of a hypochromic and not a bathochromic effect for the **AP** series suggested a different type of aromatic stacking interaction compared to **DP77** (DNA intercalator). There are several possible explanations for the observed hypochromic effect, including the intramolecular stacking of DBTAA with adenine or the intermolecular stacking of two DBTAA chromophores within DNA/RNA grooves, a solvatochromic effect in the DNA/RNA binding site, and even a weak partial intercalation of DBTAA. However, for the accurate elucidation of the precise mechanism, knowledge of the DNA/RNA binding mode is a prerequisite.

Processing of the titration data by means of the Scatchard equation [17–18] gave the binding constants and the density of the binding sites (Table 2, log *K*_s_ and ratio *n*, respectively). Data for the **APH** are not presented in the Table 2 due to the formation of the agglomerates, which cause baseline increases, thus preventing the collection of a sufficient number of data (>10 points necessary) for an accurate non-linear fitting.

**Table 2:** Binding constants (log *K*_s_)^a^ and ratios *n*^b^ ([bound compound]/[polynucleotide phosphate]) calculated from the UV–vis titrations of **AP3**, **AP3am, AP5** and **AP6** with ds-polynucleotides at pH 7.0 (buffer sodium cacodylate, *I* = 0.05 mol dm^−3^).

	**AP3**			**AP3am**			**AP5**			**AP6**		
	H^c^	log *K*_s_	*n*	H^c^	log *K*_s_	*n*	H^c^	log *K*_s_	*n*	H^c^	log *K*_s_	*n*

poly A– poly U	53	6.0	0.6	35	5.8	0.5	44	6.6	0.7	64	5.7	0.7
poly dA– poly dT	36	6.2	0.4	40	5.9	0.3	40	5.4	0.5	37	5.0	0.7
poly dAdT– poly dAdT	35	6.1	0.5	30	5.5	0.6	–^d^	–^d^	–^d^	–^d^	–^d^	–^d^
poly dG– poly dC	64	5.5	0.5	35	5.7	0.5	61	5.8	0.6	74	5.6	0.5

^a^Titration data were processed according to the Scatchard equation [17–18]; ^b^Accuracy of *n* ± 10–30%, consequently log *K*_s_ values vary in the same order of magnitude; ^c^H/% = (Abs(**APH**, **AP3**, **AP3am**, **AP5**, **AP6**) − Abs(complex))/Abs(**AP3**, **AP3am**, **AP5**, **AP6**)) × 100; ^d^Accurate calculation of log *K*_s_ values was hampered by precipitation during titration.

Examination of the data in Table 2 indicates that all studied compounds showed similar affinity toward ds-DNA and ds-RNA. The density of the binding sites (Table 2, ratio *n*) is mostly too high for the intercalative mode of binding (*n*_intercal. teor._ < 0.25). This hints at binding within the grooves of DNA/RNA or agglomeration along the DNA/RNA double helix. The possibility of several simultaneous binding modes is excluded by the isoelliptic points observed in CD experiments, which strongly support one dominant binding mode.

The thermal stabilization of ds-DNA/RNA is an essential feature of the intercalative binding mode, as well as for many groove binding molecules. The inability of the entire **AP** series to stabilize ds-DNA/RNA (Table 1), in combination with a considerable binding affinity (Table 2) gives rise to the question of the **AP** series binding mode. To shed more light on the unusual binding process of the **AP** dyes, it was necessary to assess the structure of the **AP** dye/polynucleotide complex. Application of the most informative methods like NMR and X-ray crystallography was hampered by the tendency of the **AP** dye/DNA complexes to form colloidal systems at *c* > 0.1 mM, which neither crystalized nor were suitable for NMR studies due to the extensive broadening and decreasing of proton signals, which eventually merged with the baseline (details about unsuccessful NMR experiments see [11]).

#### Circular dichroism (CD) experiments

CD spectropolarimetry offers unique possibilities for the investigation of small molecule–DNA/RNA interactions. The polynucleotide secondary structure is chiral itself, and any binding-induced conformational changes are reflected in the CD spectrum [19]. Moreover, the uniform binding of achiral small molecules within chiral DNA/RNA helix results in an induced CD spectrum (ICD) of the small molecule chromophore, whereby the ICD spectrum range >300 nm (at which DNA/RNA do not absorb) is highly informative about the orientation of the chromophore with respect to DNA/RNA chiral axis [19–20]. For instance, the intercalation of an aromatic moiety inside DNA/RNA should result in a weak negative ICD, groove binding would give a strong positive ICD, while the eventual aromatic-dimer formation within polynucleotide grooves should yield the bisignate coupled exciton electronic coupling (EC) ICD bands [20].

The studied DBTAA derivatives are achiral and are not associated with intrinsic CD spectra. Thus, the appearance of ICD bands >300 nm upon DNA/RNA binding could be used to estimate the orientation of the DBTAA chromophore in the DNA/RNA binding site.

The addition of **APH**, **AP3**, **AP3am**, **AP5** and **AP6** resulted in a decrease of the ds-DNA/RNA CD bands (range from 220 to 300 nm, Supporting Information File 1), whereas the isoelliptic points supported the formation of only one type of the compound/DNA or RNA complex. The intensity decrease of ds-DNA/RNA CD bands is usually associated with the partial disruption of the polynucleotide helical chirality caused by the binding of a small molecule.

**Figure 2:**
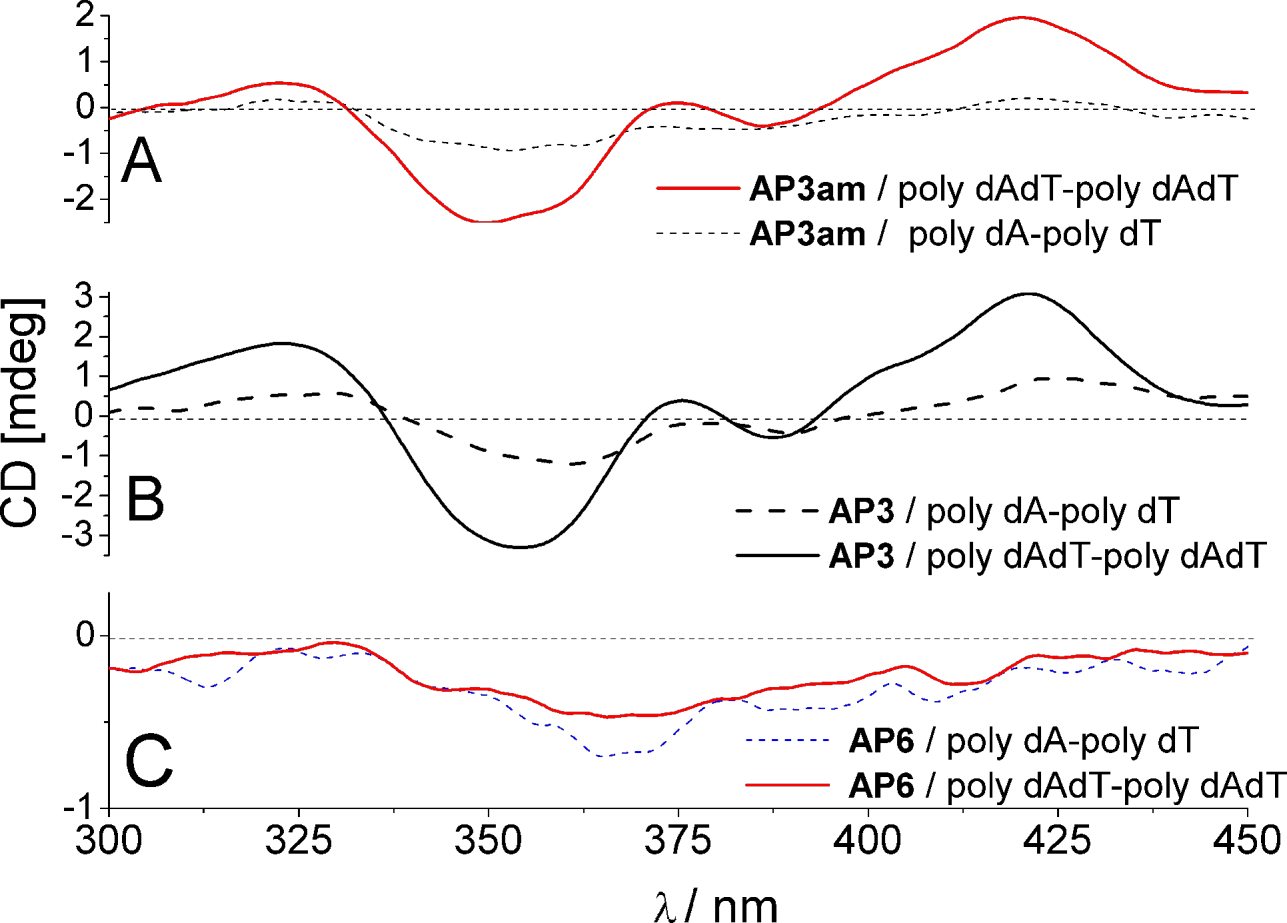
Induced CD bands observed for **AP3**, **AP3am**, **AP6** for poly dA–poly dT and poly (dAdT)_2_ (*c* = 3.0 × 10^−5^ mol dm^−3^). The dG–dC and rA–rU polynucleotides did not give any induced (I)CD bands >300 nm.

The reference compound **APH**, as well as **AP5** and **AP6** (Figure 2C) with all studied ds-DNA/RNA give negligible (if any) ICD bands in the range of >300–450 nm, which along with the moderate affinity (Table 2) and the absence of thermal stabilization (Table 1), suggested a non-specific aggregation of molecules along polynucleotides. Such an unspecific binding mode (possible locations are minor/major groove, not excluding weak partial intercalation of the DBTAA moiety) results in DBTAA chromophores characterized by a variety of orientations oriented variously in respect to the DNA/RNA chiral axis, which can yield the observed weak non-descriptive ICD pattern [19–20].

By contrast with **APH**/**AP5**/**AP6**, the “propyl” analogues **AP3** and **AP3am** revealed significantly different ICD patterns >300 nm. Strongly induced ICD bands were observed only for AT-containing DNAs (Figure 2A,B), but not for GC-DNA and ds-RNA (Supporting Information File 1). Intriguingly, the ICD pattern observed for **AP3** and **AP3am** (Figure 2A,B; positive–negative–positive sign of ICD bands) were distinctively different from the very weak negative ICD bands of **AP5** (Supporting Information File 1) and **AP6** (Figure 2C). Moreover, save for **AP3**, **AP3am** gave ICD bands (Supporting Information File 1) with mixed sequence ct-DNA (58% AT base pairs).

Although **AP3** and **AP3am** ICD spectra with AT-DNA closely resemble the ICD spectrum of the previously studied **DP77** (no adenine attached to DBTAA), there are several differences between **AP3/AP3am** and **DP77**. Only **DP77** [12] gives an ICD spectrum for ds-RNA (poly rA–poly rU) and ICD at low ratios_[compd]/[DNA]_ (0.1; 0.2), which is attributed to **DP77–DBTAA** intercalation. Under the same conditions the ICD bands of **AP3/AP3am** exhibit a significant intensity only at r > 0.3, which strongly corroborates the hypothesis of the dimerization of the DBTAA chromophore within the DNA groove [20]. A more detailed analysis of ICD data revealed that ICD bands of **AP3/AP3am** were highly sensitive to the secondary structure of DNA/RNA and, in particular, the minor groove properties (Table S1, Supporting Information File 1). For instance, the ICD bands of **AP3/AP3am** show the strongest intensity for an alternating AT–AT-polynucleotide characterized by a minor groove size of 6.3 Å, which is ideal for the accommodation of an aromatic dimer (VdW sum of stacked aromatics ~7 Å, Figure 4). In contrast to alternating-AT, the homo-polynucleotide poly dA–poly dT minor groove is much narrower (3.3 Å), and the lower intensity of bisignate ICD bands points to a lower percentage of dimer formation (due to the necessary unwinding and adaptation of homo-polynucleotide to accommodate a DBTAA-dimer). Homo- and alternating-AT-DNA also significantly differ in terms of the charge density and the curvature of the DNA backbone, which can influence interactions of the positively charged arm of **AP3/AP3am** with the phosphate backbone and **AP3/AP3am**-adenine with thymines of the DNA.

The GC-DNA and AU-RNA minor grooves are not suitable for an aromatic dimer binding. In the case of GC-DNA this is due to the steric hindrance of the guanine amino groups inside the groove. In the case of the AU-RNA, it is because of the very wide and shallow shape, which does not support the binding of a small molecule (Table S1, Supporting Information File 1). This is consistent with the fact that no **AP3/AP3am** ICD bands were observed for GC-DNA and ds-RNA.

**Figure 3:**
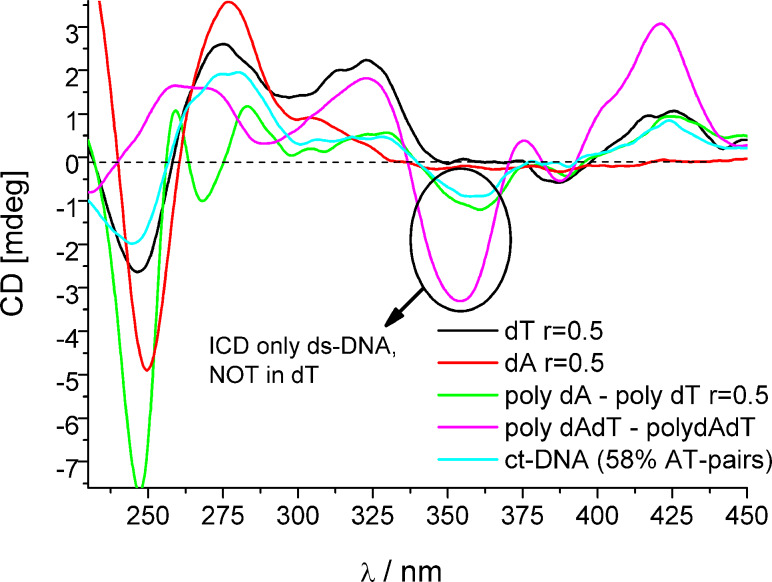
Comparison of **AP3** ICD bands for ss-DNA (dA and dT) with alternating- and homo-AT ds-polynucleotides, as well as mixed sequence DNA (ct-DNA, 58% of AT).

In a previous study **AP3** revealed an unprecedented recognition of a dT sequence by the appearance of a specific ICD band, while it did not exhibit an ICD signal for other ss-DNA/RNA [11]. A comparison of ICD bands for various ss- and ds-DNA (Figure 3) revealed that the strong negative band at 350 nm is characteristic for ds-DNA, whereas the positive bands at 323 and 421 nm are common for both, ss-dT and ds-AT-DNA. Thus, the negative ICD band at 350 nm can be assigned to a part of the bisignate coupled exciton electronic coupling (EC) ICD band of the **AP3** (also **AP3am**) dimer inside the ds-DNA minor groove. On the other hand, **AP3** binds to poly dT as a single molecule (not stacked with other DBTAA) and, therefore, it is lacking the characteristic bisignate ICD band at 350 nm. Interestingly, the negative ICD band at 350 nm is also visible for ct-DNA (58% AT base pairs), which confirms that any AT-DNA sequence even in mixed polynucleotides will give a characteristic ICD pattern.

#### Structure–activity relations of DBTAA–adenine conjugates in AT-DNA binding

**Figure 4:**
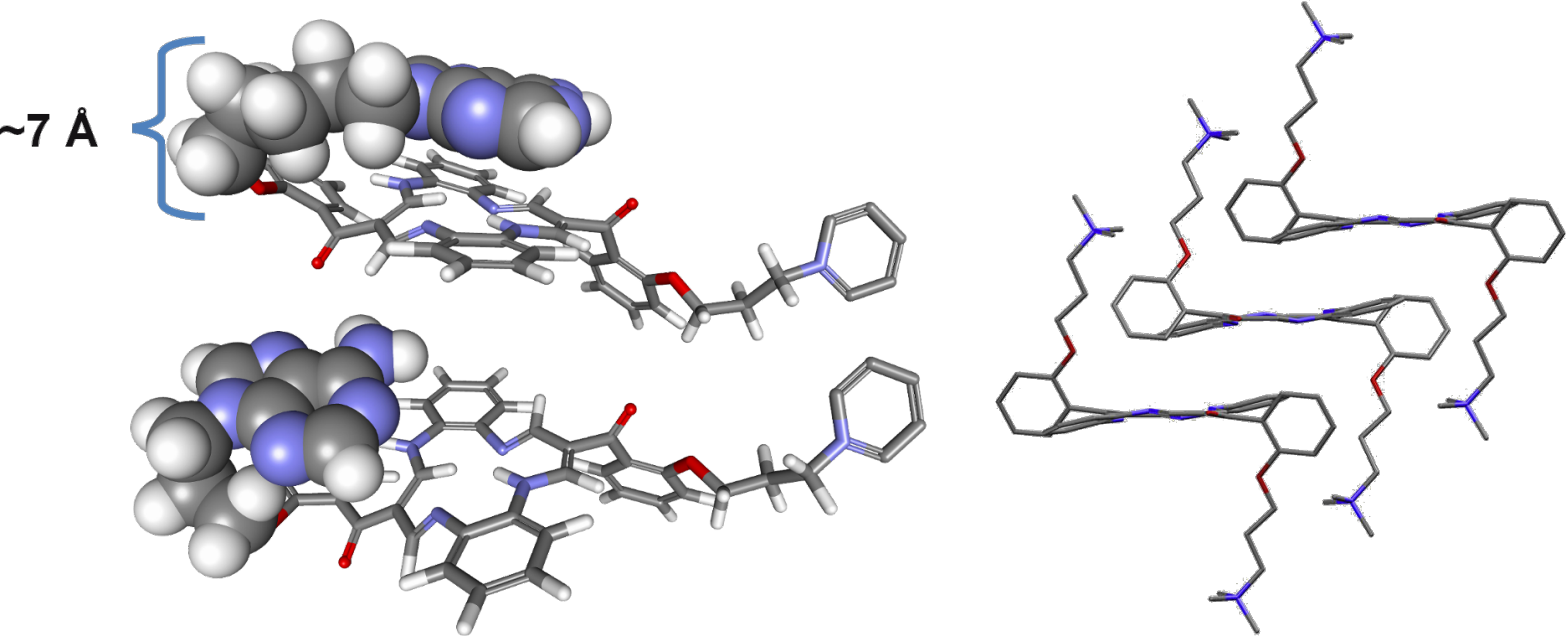
Self-folding of **AP5** (upper left – note the stacking of DBTAA and adenine) and **AP3** (lower left – note the short linker which does not allow for the stacking of DBTAA and adenine); (right) detail of three molecules packing in crystal by π–π stacking of parallel DBTAA rings with a distance of ca. 3 Å (see [13] and Figure 8 therein).

Despite the results described above, it is still not clear whether the origin of the DBTAA-ICD pattern proposed for the AT-DNA recognition is an intermolecularly stacked system formed by two DBTAA moieties or, alternatively, a single intramolecularly stacked DBTAA–adenine molecule. The solubility of the system prevented NMR experiments. So we relied on structural differences (linker length connecting DBTAA and adenine) between **AP3**/**AP3am** and **AP5**/**AP6** as free molecules for our proposal of the most likely structure explanation. The chosen derivatives (**AP5** and **AP3**) were constructed by starting from the crystal structure of their close analogue [13], and then manually self-folded to bring adenine on top of DBTAA. Finally, their chemical bond lengths and angles were corrected by the MM2-optimization in the software MarvinSuite to assure a realistic presentation (Figure 4). These models serve as a schematic presentation for an easier discussion of the experimental results.

**Figure 5:**
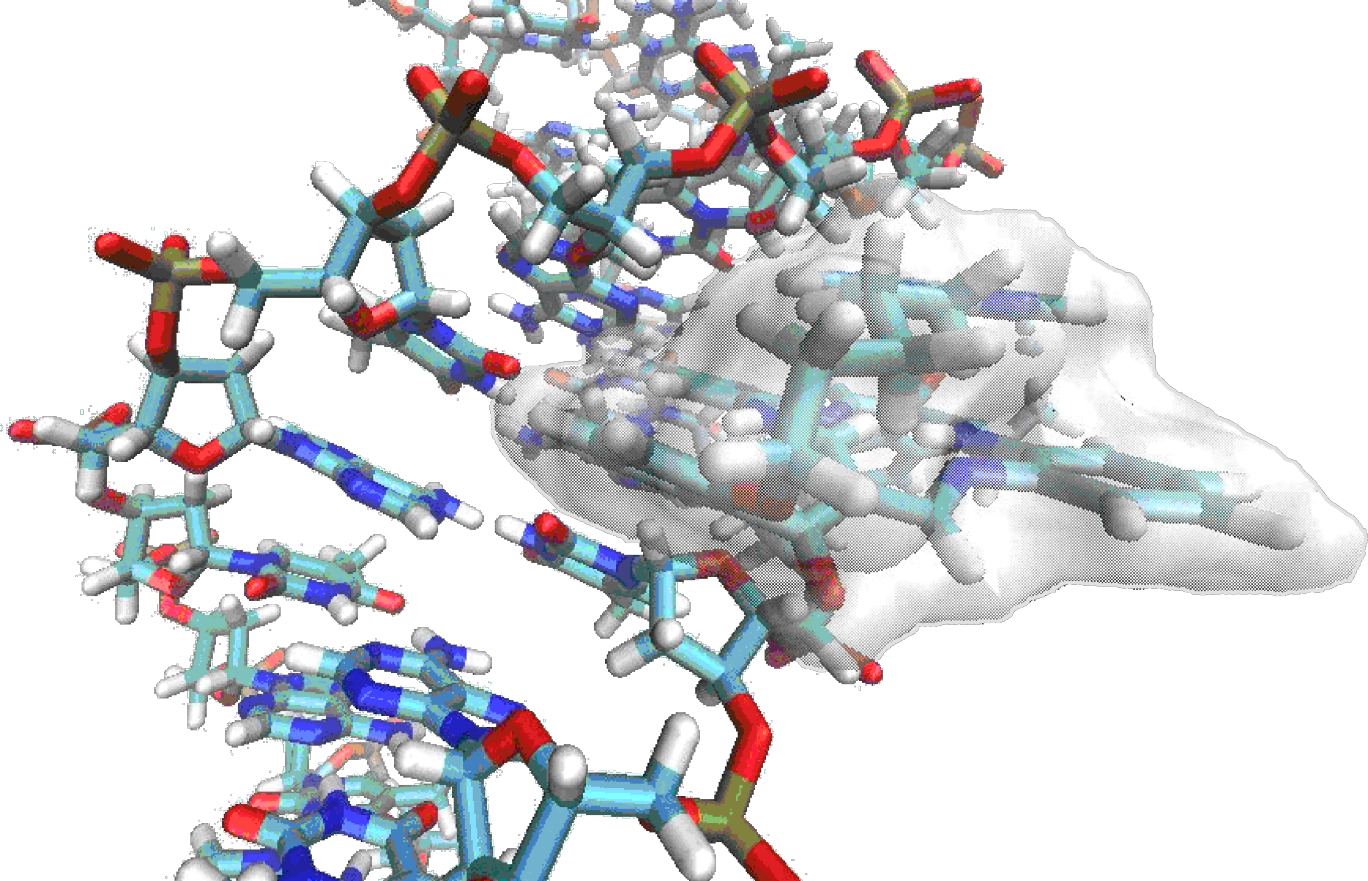
Schematic presentation of self-folded **AP5** (free **AP5** see Figure 4; upper left) in the minor groove of poly dAdT–poly dAdT. Double stranded DNA (previously used in [6]) was created with the program NUCGEN, a part of the AMBER11 program suite [21]. The self-folded ligand **AP5** was docked manually into the poly (dA–dT)_2_ polynucleotide minor groove by using the program VMD [22], taking into account that VdW radii of DNA and ligand do not overlap.

The **AP3**/**AP3am** cannot form efficient intramolecular stacks because the **AP3**/**AP3am** propyl linker is too short to allow adenine to stack above the DBTAA moiety (Figure 4 lower left). Thus, it can be expected that, given an excess of **AP3** over DNA binding sites (r > 0.3), two **AP3**-DBTAA moieties will form π–π stacked dimers within the DNA minor groove similar to the crystal structure of close analogue (Figure 4, right). Conversely, the **AP5** and **AP6** aliphatic linkers are long enough to allow self-folding and easy intramolecular π–π stacking of adenine with DBTAA (Figure 4, upper right). During the binding event to ds-DNA (AT minor groove) **AP5** and **AP6** have to combine intramolecular (self-folded) and intermolecular (with DNA) interactions, whereby the best compromise could be the binding of self-folded molecules within a DNA minor groove (see schematic presentation in Figure 5).

The reference compound **APH** has only one side arm interacting with DNA so that a number of orientation options upon DNA/RNA binding is increased, which results in a negligible/non-descriptive ICD pattern. It should be stressed that the absence of **APH**-ICD bands emphasizes the importance of adenine in **AP3/AP3am** for the induction of AT-DNA ICD pattern.

## Conclusion

The **AP** derivatives presented herein are found to non-covalently bind to ds-DNA/RNA. The absence of bathochromic shifts in UV–vis titrations, the lack of a thermal stabilization effect on any ds-DNA/RNA, as well as the previously reported [11] viscometry and gel electrophoresis results do not support intercalation as the dominant binding mode (not excluding weak partial intercalation of the DBTAA moiety in some cases). Intrinsically achiral, **AP**-derivatives reveal significantly different induced CD bands upon binding to ds-DNA/RNA. Only DBTAA–propyladenine conjugates (**AP3/AP3am**) revealed molecular recognition of AT-DNA by the appearance of an ICD band pattern >300 nm. Significant ICD bands did not appear for other ds-DNA/RNA. A structure–activity relation for the studied series of compounds revealed that the essential structural features for the ICD recognition of AT-DNA are a) the presence of DNA-binding appendages on both DBTAA side chains, i.e., the adenine side chain and the positively charged side chain and b) the presence of a short propyl linker, which does not support intramolecular aromatic stacking between DBTAA and adenine.

The essential difference between **AP3**/**AP3am** and **DP77** is that the former give ICD bands exclusively for AT-DNA, while the latter gives various ICD patterns for all ds-DNA and even ds-RNA. Thus **DP77** is not suitable for the recognition of AT-DNA.

Furthermore, the ICD patterns of **AP3** with ss-DNA (oligo dT) and ds-AT-DNA essentially differ in the appearance of a strong negative band at 350 nm, characteristic for ds-DNA. This band could be used to probe the efficiency of pairing oligo dT sequences with complementary dA or rA structures, for instance, in any (antisense strategy) developed oligonucleotide, which relies on efficient and precise AT sequence pairing. Namely, the addition of **AP3**/**AP3am** to ss-DNA containing an oligo dT sequence should yield the specific ICD pattern of dT among any other ss-DNA/RNA sequence present in solution [11]. The ICD pattern upon the addition of the exclusively matching oligo A sequence should change by the appearance of a strong negative band at 350 nm, which is characteristic for ds-AT-DNA. Another application could be related to oligo (dT)-cellulose commonly used for tRNA purification [23]. In particular, the difference in **AP3**/**AP3am** ICD pattern between cellulose-tagged dT and coupled double-stranded oligo dT–rA can be reported and its stability and structure studied in detail by monitoring the changes in ICD shape and intensity.

Furthermore, DBTAA–derivatives are azamacrocyclic ligands and thus have additional potential to bind metal cations [24], such metal complexes offering a variety of different interactions with DNA/RNA. Future prospects also include the synthesis of fluorescent DBTAA derivatives, which are expected to transfer observed intriguing DBTAA-DNA/RNA binding properties in a lower concentration range and broaden the set of DBTAA applications by cell-bioimaging. Such applications are also supported by the preliminary bioactivity screening of the AP-series, which revealed low cytotoxicity toward human cell lines.

## Experimental

### General

7,16-Bis[2-(3-bromopropoxy)benzoyl]-5,14-dihydrodibenzo[*b*,*i*][1,4,8,11]tetraazacyclotetradecine, 7,16-Bis[2-hydroxybenzoyl]-5,14-dihydrodibenzo[*b*,*i*][1,4,8,11]tetraazacyclotetradecine and 9-(6-bromopentyl)adenine were prepared by the procedure described earlier [13–15]. All reagents were purchased from commercial sources (Sigma-Aldrich) and were used as received. Solvents were dried by using standard methods and were freshly distilled before use.

^1^H and ^13^C NMR were run on Bruker AVANCE II 300 and Bruker AVANCE III 600 spectrometers. Chemical shifts (δ) are expressed in parts per million and J values in hertz. Signal multiplicities are denoted as s (singlet), d (doublet), t (triplet), q (quartet), and m (multiplet). The IR-ATR spectra were recorded with a Thermo Fisher Scientific Nicolet IR200. ESI mass spectra were taken on a Bruker Daltonics microTOF-II spectrometer.

### Syntheses

**(7-{2-[3-(Adenin-9-yl)propoxy]benzoyl}-16-{2-[3-(*N*,*N*,*N*-trimethylammonium)propoxy]benzoyl}-5,14-dihydrodibenzo[*b*,*i*][1,4,8,11]tetraazacyclotetradecine bromide) (AP3am):** Similar as described in [11] a mixture consisting of adenine (0.175 g, 1.298 mmol) and 60% NaH (0.039 g, 0.931 mmol) in anhydrous DMF (3 mL) was stirred for 1 h at room temperature. 7,16-Bis[2-(3-bromopropoxy)benzoyl]-5,14-dihydrodibenzo[*b*,*i*][1,4,8,11]tetraazacyclotetradecine [13] (0.5 g, 0.649 mmol) dissolved in hot anhydrous DMF (40 mL) was added, and the reaction mixture was stirred at 40 °C for 5 min, then for 3 h at room temperature. The reaction mixture was then cooled in a freezer and partitioned between dichloromethane (70 mL) and water (100 mL). The organic layer was separated, washed with water (2 × 100 mL), dried over anhydrous magnesium sulfate, concentrated to a small volume, and chromatographed on a column of silica gel with dichloromethane/methanol (20:0.5 to 20:1 v/v) as an eluent. The main orange fraction was collected and evaporated to dryness. A residue was dissolved in DMF (3 mL) and placed in an ice bath. 0.6 mL of cooled trimethylamine was added, and the mixture was stirred for 5 h at 50 °C. The excess of trimethylamine was removed under diminished pressure, and 30 mL of *tert*-butyl methyl ether was added. Crystallized solid was collected, washed with *tert*-butyl methyl ether, dried in vacuum, and chromatographed on a column with basic aluminum oxide with dichloromethane/methanol (20:0.5 to 20:2 v/v) as an eluent. The main orange fraction was collected, evaporated to dryness, and dissolved in methanol (2 mL). An orange-red microcrystalline product was obtained by the slow diffusion of diethyl ether and the drying isolated solid under vacuum. Yield: 0.046 g (8%). ^1^H NMR (600 MHz, DMSO-*d*_6_) δ (ppm) 14.29 (m, 1H, H^10^/H^21^), 14.20 (m, 1H, H^10^/H^21^), 8.55 (d, *J* = 6.7 Hz, 2H, {H^7^, H^9^}/{H^18^, H^20^}), 8.43 (d, *J* = 6.5 Hz, 2H, {H^7^, H^9^}/{H^18^, H^20^}), 7.91 (s, 1H, H^h^), 7.86 (s, 1H, H^k^), 7.52 (m, 2H, H^27^, H^27'^), 7.39 (m, 2H, H^25^, H^25'^), 7.09-7.31 (m, 12H, H^1^-H^4^, H^12^-H^15^, H^26^, H^26'^, H^28^, H^28'^), 7.07 (s, 2H, H^m^), 4.09–4.14 (m, 4H, H^g^, H^a^), 4.03 (t, *J* = 5.9 Hz, 2H, H^e^), 3.28 (m, 2H, H^c^), 2.92 (s, 9H, H^d^), 2.11 (m, 2H, H^f^), 2.03 (m, 2H, H^b^); ^13^C NMR (75 MHz, DMSO-*d*_6_) δ (ppm) 191.2, 191.4 (C^23^, C^23'^), 155.7 (C^l^), 154.8, 155.0 (C^29^, C^29'^), 152.3, 152.7 (C^7^, C^9^, C^18^, C^22^), 152.1 (C^k^), 149.2 (C^j^), 140.4 (C^h^), 136.1, 136.3 (C^5^, C^11^, C^16^, C^22^), 131.4 (C^27^, C^27'^), 129.1, 129.2 (C^25^, C^25'^), 128.7, 128.8 (C^24^, C^24'^), 126.7 (C^2^, C^3^, C^13^, C^14^), 120.9, 121.2 (C^26^, C^26'^), 118.6 (C^i^), 115.3, 115.5 (C^1^, C^4^, C^12^, C^15^), 112.7, 113.0 (C^28^, C^28'^), 110.0, 110.2 (C^18^, C^19^), 65.1 (C^a^, C^e^), 62.8 (C^c^), 52.0 (C^d^), 29.0, 22.5 (C^b^, C^f^); ESI-HRMS (*m/z*): M^+^ calcd for C_46_H_47_N_10_O_4_, 803.378; found, 803.376; IR-ATR (cm^−1^): 3525, 3374, 3314, 3177, 3083, 2959, 2919, 2868, 1651, 1600, 1562, 1484, 1449, 1417, 1396, 1286, 1263, 1239, 1219.

**(7-{2-[5-(Adenin-9-yl)pentoxy]benzoyl}-16-{2-[5-(*N*-pyridinium-1-yl)pentoxy]benzoyl}-5,14-dihydrodibenzo[*b*,*i*][1,4,8,11]tetraazacyclotetradecine bromide) (AP5):** Similar as described in [11] a reaction mixture consisting of 7,16-Bis[2-hydroxybenzoyl]-5,14-dihydrodibenzo[*b*,*i*][1,4,8,11]tetraazacyclotetradecine [14] (0.2 g, 0.378 mmol), anhydrous potassium carbonate (0.104 g, 0.757 mmol), and 9-(6-bromopentyl)adenine [15] (0.054 g, 0.189 mmol) in anhydrous DMF (40 mL) was stirred for 72 h at room temperature. 1,5-Dibromopentane (0.618 mL, 4.54 mmol) and anhydrous potassium carbonate (0.026 g, 0.189 mmol) were then added, and the stirring was continued for 24 h at room temperature. The reaction mixture was partitioned between dichloromethane (20 mL) and water (100 mL). A small amount of solid KBr was added to improve the separation of the phases. The organic layer was separated and washed thoroughly with water (5 × 30 mL), dried over anhydrous magnesium sulfate, concentrated to a small volume, and chromatographed on a column of silica gel with dichloromethane/methanol (10:0.6 v/v) as an eluent. The second fraction was collected from the two orange ones which displayed the highest intensity. It was evaporated to dryness, dissolved in 3 mL of chloroform, and, once again, chromatographed on a column of silica gel with chloroform/methanol (20:0.4 to 20:0.6 v/v) as an eluent. The main fraction was collected, evaporated to dryness, and a solid residue was dissolved in pyridine (5 mL). The mixture was stirred for 7 h at 45 °C, then pyridine was removed under diminished pressure, and the solid residue was chromatographed on a column with basic aluminum oxide with dichloromethane/methanol (20:0.5 to 20:3 v/v) as an eluent. The main orange fraction was collected and evaporated to dryness. An orange-red microcrystalline product was obtained by drying under vacuum. Yield: 0.035 g (19%). ^1^H NMR (300 MHz, DMSO-*d*_6_) δ (ppm) 14.26 (m, 2H, H^10^, H^21^), 8.95 (m, 2H, H^f^), 8.54 (tt, *J* = 7.8 Hz, *J* = 1.3 Hz, 1H, H^h^), 8.45 (m, 4H, H^7^, H^9^, H^18^, H^20^), 8.05 (m, 2H, H^g^), 7.95 (s, 1H, H^n^), 7.86 (s, 1H, H^q^), 7.50 (m, 2H, H^27^, H^27'^), 7.33 (dd, *J* = 3.7 Hz, *J* = 1.8 Hz, 1H, H^25^/H^25'^), 7.04–7.26 (m, 15H, H^1^-H^4^, H^12^-H^15^, H^25^/H^25'^, H^26^, H^26'^, H^28^, H^28'^, H^s^), 4.43 (t, *J* = 7.6 Hz, 2H, H^e^), 4.00 (m, 4H, H^a^, H^m^), 3.87 (t, *J* = 7.3 Hz, 2H, H^i^), 1.78 (m, 2H, aliphatic chain), 1.59 (m, 6H, aliphatic chain), 1.24 (m, 4H, aliphatic chain); ^13^C NMR (75 MHz, DMSO-*d*_6_) δ (ppm) 191.3, 191.4 (C^23^, C^23'^), 155.7 (C^r^), 155.1, 155.1 (C^29^, C^29'^), 152.4, 152.5 (C^7^, C^9^, C^18^, C^20^), 152.1 (C^q^), 149.3 (C^p^), 145.3 (C^h^), 144.4 (C^f^), 140.2 (C^n^), 136.1 (C^5^, C^11^, C^16^, C^22^), 131.3, 131.5 (C^27^, C^27'^), 129.0, 129.2 (C^25^, C^25'^), 128.6 (C^24^, C^24'^), 127.9 (C^g^), 126.7 (C^2^, C^3^, C^13^, C^14^), 120.7 (C^26^, C^26'^), 118.5 (C^o^), 115.2 (C^1^, C^4^, C^12^, C^15^), 112.5, 112.5 (C^28^, C^28'^), 109.9, 110.0 (C^8^, C^19^), 67.5, 67.5 (C^a^, C^i^), 60.3 (C^e^), 42.4 (C^m^), 21.8, 22.4, 27.6, 27.8, 28.9, 30.1 (C^b^–C^d^, C^j^–C^l^); ESI-HRMS (*m/z*): M^+^ calcd for C_52_H_51_N_10_O_4_, 879.409; found, 879.407; IR-ATR (cm^−1^): 1249, 1286, 1412, 1446, 1483, 1560, 1588, 1643, 2863, 2935, 3057, 3317.

### Spectrophotometric studies

The UV–vis spectra were recorded on a Varian Cary 100 Bio spectrophotometer and the CD spectra on a JASCO J815 spectrophotometer at 25 °C with appropriate 1 cm path quartz cuvettes. The study of interactions with DNA and RNA was carried out with aqueous solutions of compounds buffered to pH 7.0 (buffer sodium cacodylate, *I* = 0.05 mol dm^−3^).

Polynucleotides were purchased as noted: poly dAdT–poly dAdT, poly dG–poly dC, poly dA–poly dT, poly A–poly U (Sigma-Aldrich, St. Louis. USA), calf thymus (ct)-DNA (Aldrich). Polynucleotides were dissolved in sodium cacodylate buffer, *I* = 0.05 mol dm^−3^, pH 7.0. The calf thymus ct-DNA was additionally sonicated and filtered through a 0.45 mm filter [25]. The polynucleotide concentration was spectroscopically determined as the concentration of nucleobases. Spectrophotometric titrations were performed at pH 7.0 (*I* = 0.05 mol dm^−3^, buffer sodium cacodylate) by adding portions of polynucleotide solution into the solution of the studied compound for UV–vis. CD experiments were carried out by adding portions of compound stock solution into the solution of polynucleotide. Titration data were processed by the Scatchard equation [17–18]. The values for *K*_s_ and *n* given in Table 2 all have satisfactory correlation coefficients (>0.999). The thermal melting curves for DNA, RNA and their complexes with the studied compounds (Table 1) were determined as previously described [16,26] by following the absorption change at 260 nm as a function of the temperature. The absorbance of the ligands was subtracted from every curve, and the absorbance scale was normalized. *T*_m_ values are the midpoints of the transition curves determined from the maximum of the first derivative and checked graphically by the tangent method [26]. The Δ*T*_m_ values were calculated by subtracting *T*_m_ of the free nucleic acid from *T*_m_ of the complex. Every reported Δ*T*_m_ value was the average of at least two measurements. The error of Δ*T*_m_ is ±0.5 °C.

## Supporting Information

File 1 Additional NMR spectra for new compounds, additional UV–vis and CD spectra.
