# Supplementary material for: Molecular recognition of AT-DNA sequences by the induced CD pattern of dibenzotetraaza[14]annulene (DBTAA)–adenine derivatives
Source: Beilstein J Org Chem. 2014 Sep 12;10:2175–85. doi: 10.3762/bjoc.10.225 (PMC4168902; doi:10.3762/bjoc.10.225)
Supplement: File 1 — Additional NMR spectra for new compounds, additional UV–vis and CD spectra. [file Beilstein_J_Org_Chem-10-2175-s001.pdf]

Supporting Information

for

Molecular recognition of AT-DNA sequences by the induced CD pattern of dibenzotetraaza[14]annulene (DBTAA)–adenine derivatives

Marijana Radić Stojković[1], Marko Škugor[1], Łukasz Dudek[2], Jarosław Grolik[2], Julita Eilmes[2] and Ivo Piantanida [1]*

Address: [1]Laboratory for Study of Interactions of Biomacromolecules, Division of Organic Chemistry and Biochemistry, Ruđer Bošković Institute, Bijenička cesta 54, PO Box 180, HR-10002 Zagreb, Croatia and [2]Department of Chemistry, Jagiellonian University, Ingardena 3 ,30-060 Kraków, Poland

Email: Ivo Piantanida - pianta@irb.hr

* Corresponding author

Additional NMR spectra for new compounds characterisation, additional UV–vis and CD spectra

^{1}H NMR and HRMS–ESI spectra of **AP3am** and **AP5**

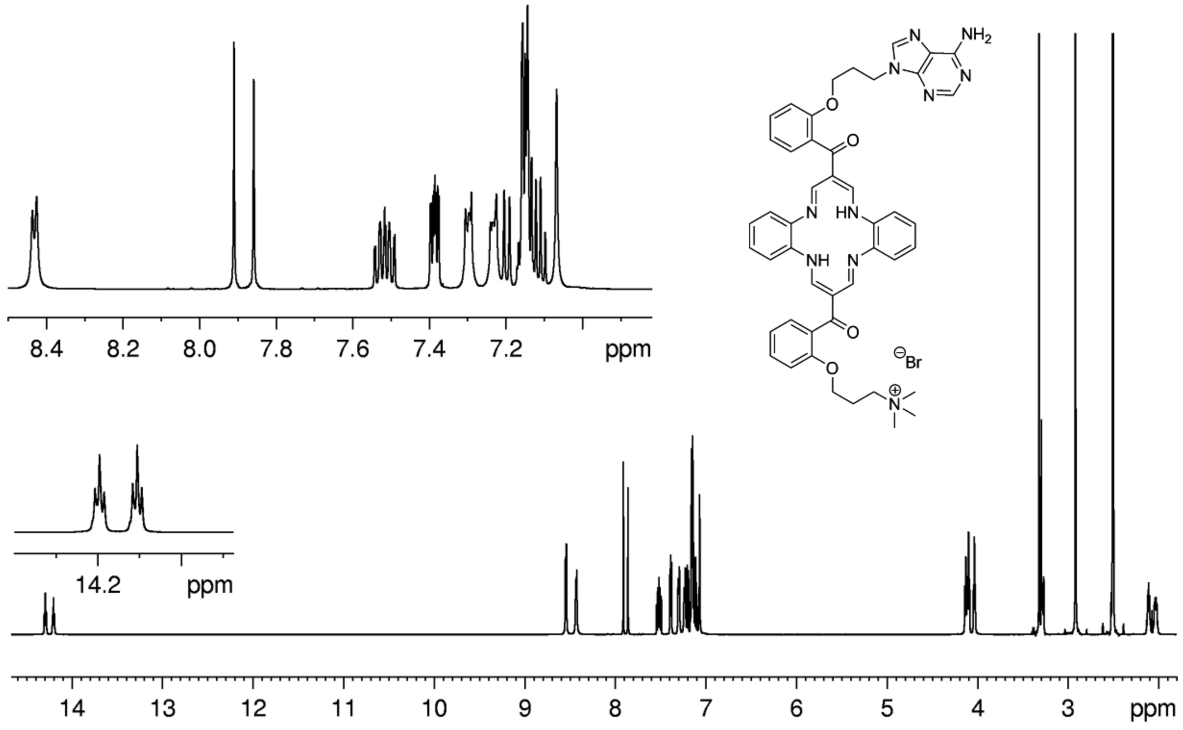

^{1}H NMR spectrum of **AP3am**

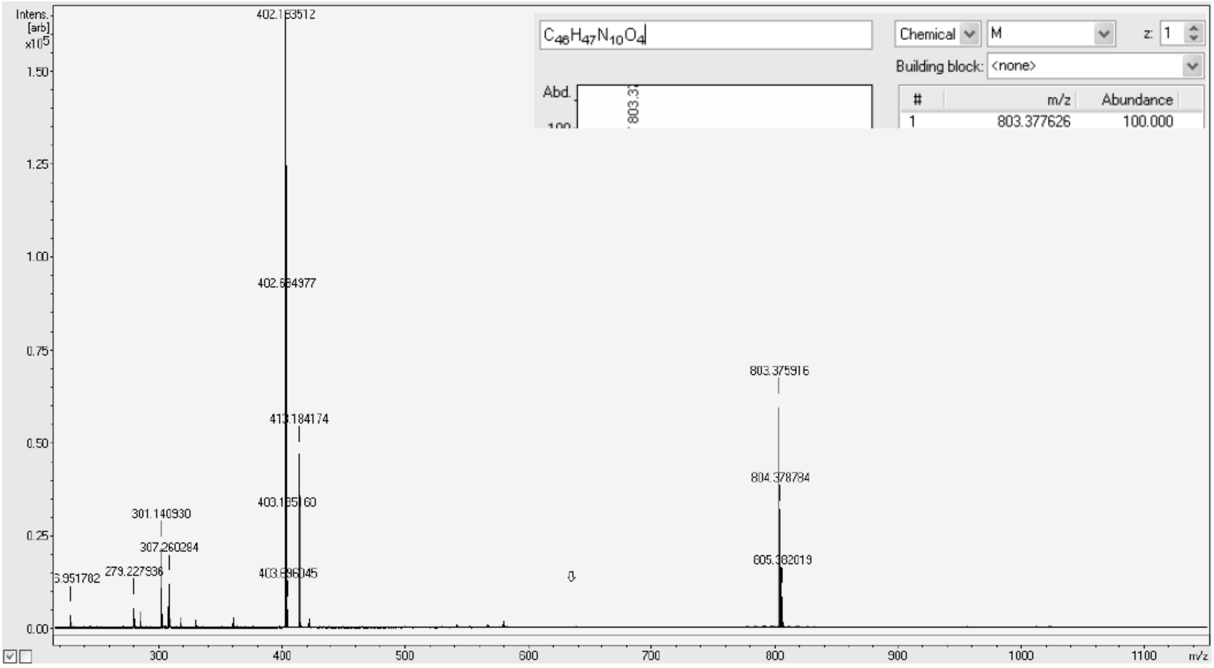

ESI-HRMS spectrum of **AP3am**

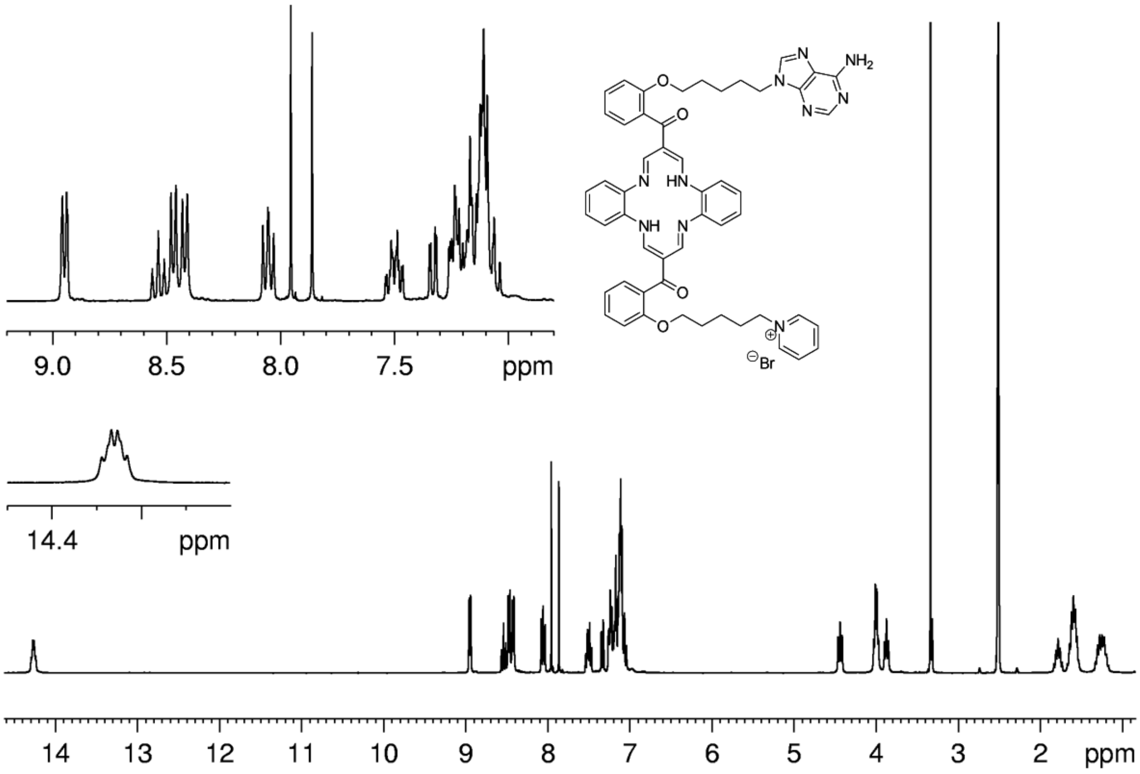

^{1}H NMR spectrum of **AP5**

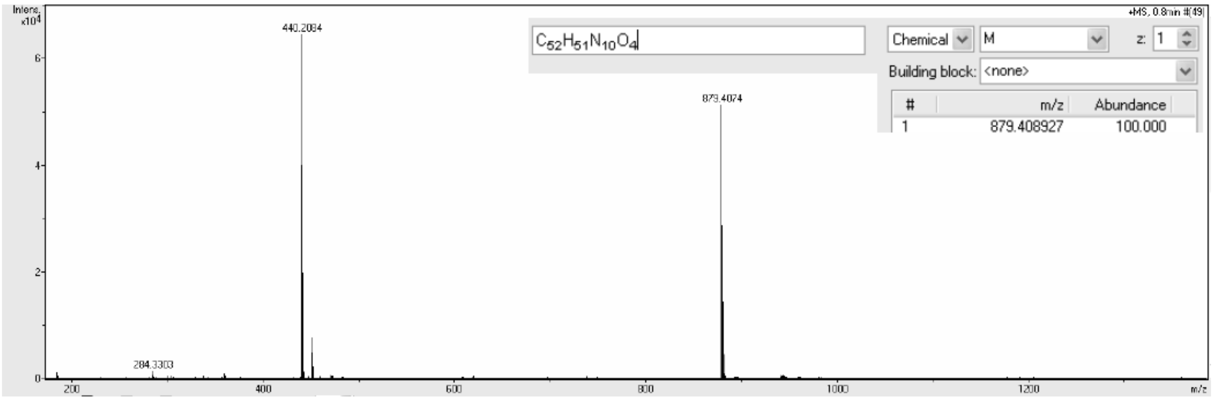

ESI-HRMS spectrum of **AP5**

UV–vis experiments:

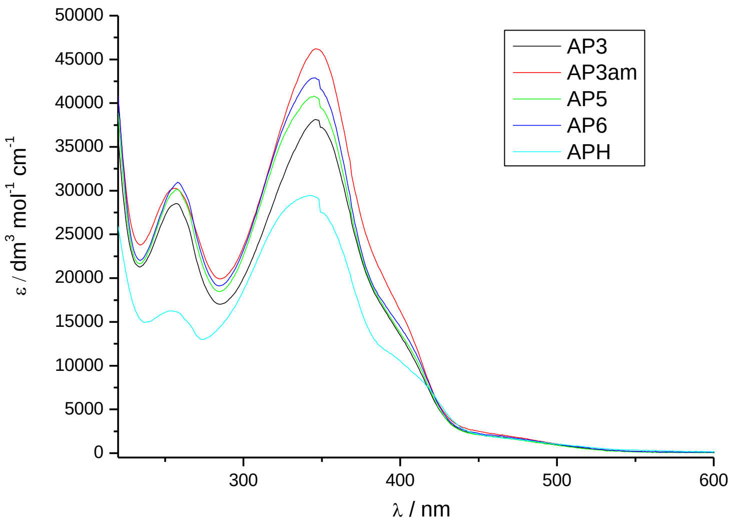

Figure S1. UV–vis spectra of **AP compounds** at c = 1.6 × 10^{-5} mol dm^{-3}; pH 7.0, sodium cacodylate/HCl buffer, *I* = 0.05 mol dm^{-3}.

CD experiments:

	ctDNA	poly dG–poly dC
**APH**	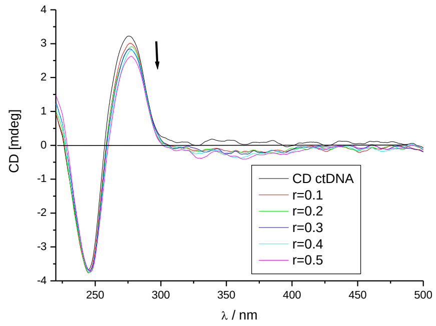	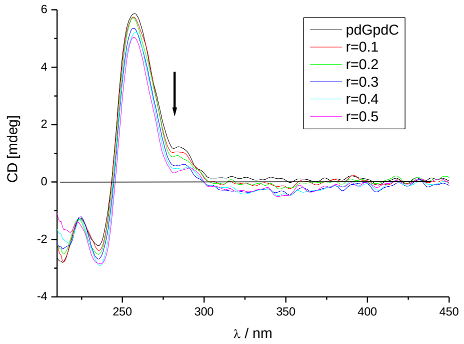
**AP3**	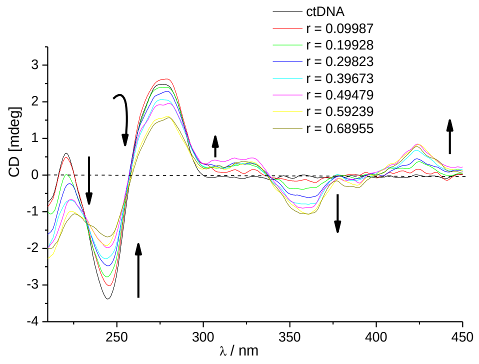	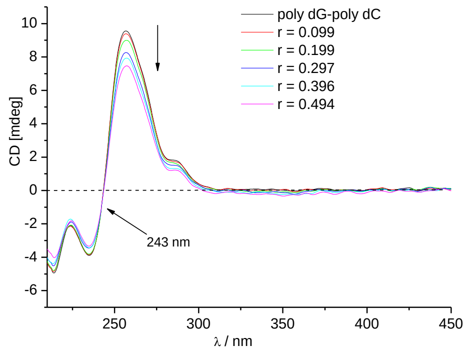
**AP3am**	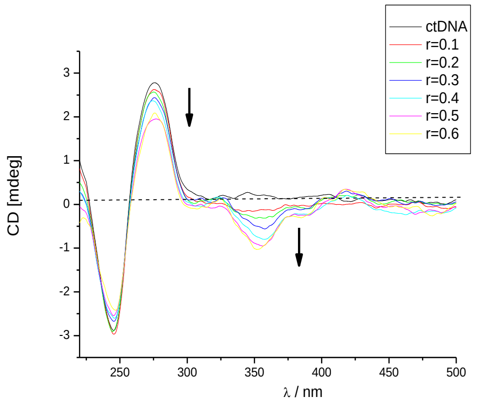	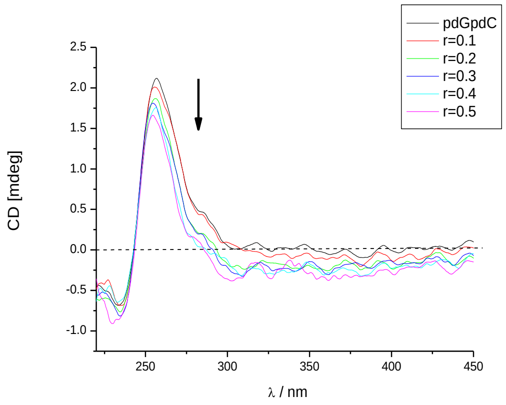

AP5

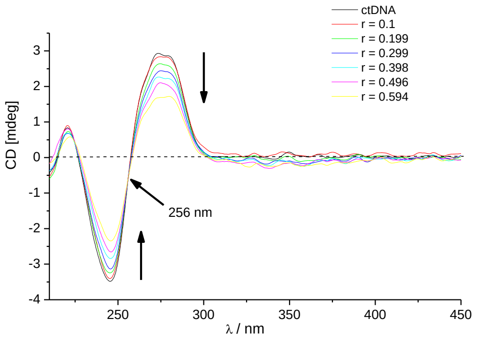

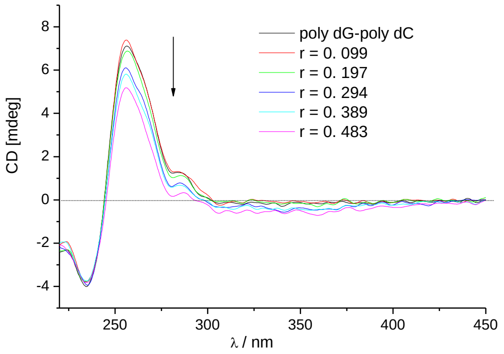

AP6

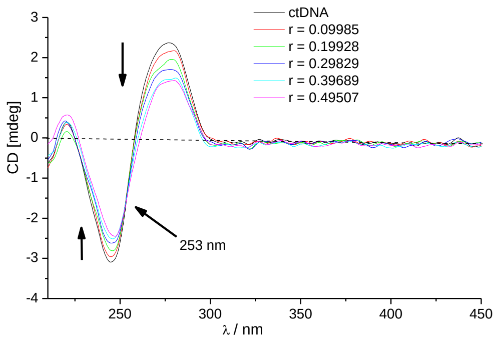

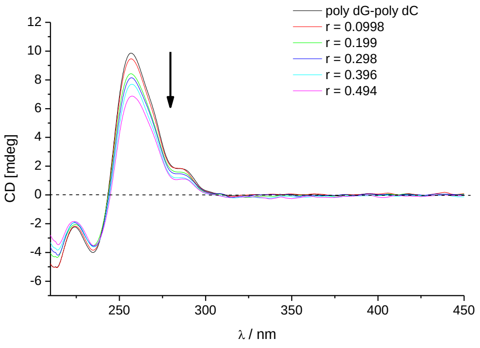

Figure S2. CD titration of **ctDNA** ($c = 3.0 \times 10^{-5}$ mol dm^{-3}) and **poly dG–poly dC** ($c = 3.0 \times 10^{-5}$ mol dm^{-3}) with **APH**, **AP3**, **AP3am, AP5** and **AP6** at molar ratios ***r*** = [compound] / [polynucleotide] (pH 7.0, buffer sodium cacodylate, I = 0.05 mol dm^{-3}).

	poly A–poly U	poly dA–poly dT
**DP77**	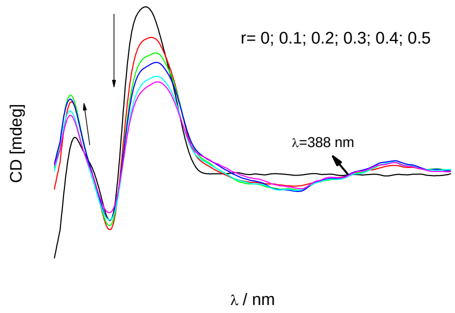	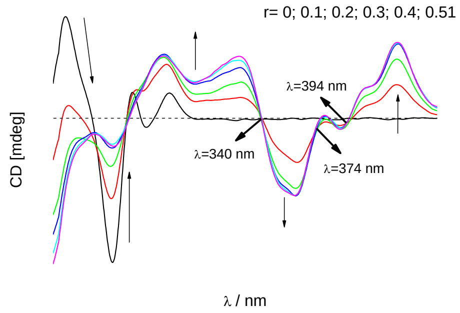
**APH**	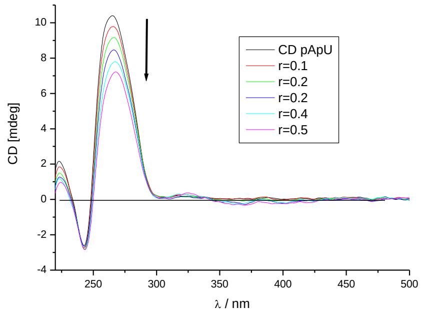	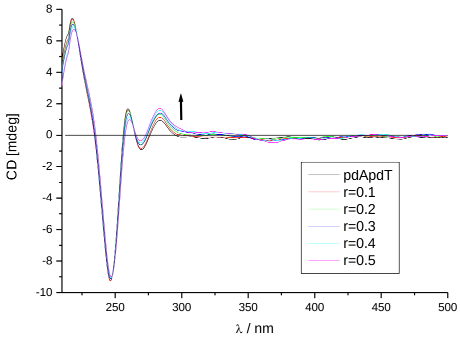
**AP3**	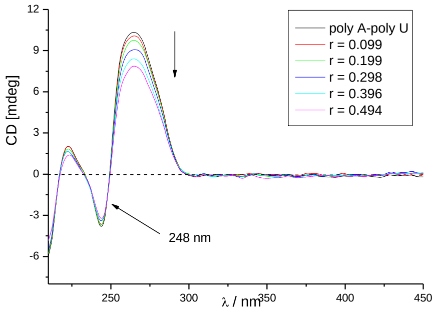	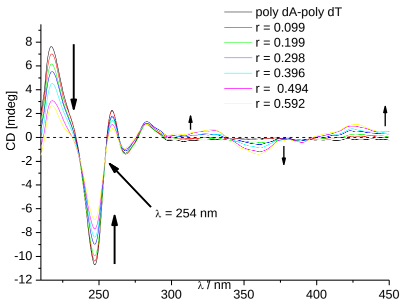
**AP3am**	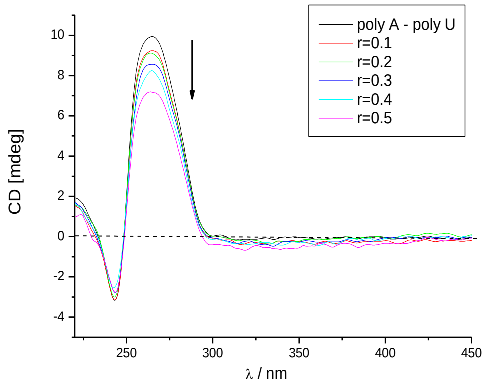	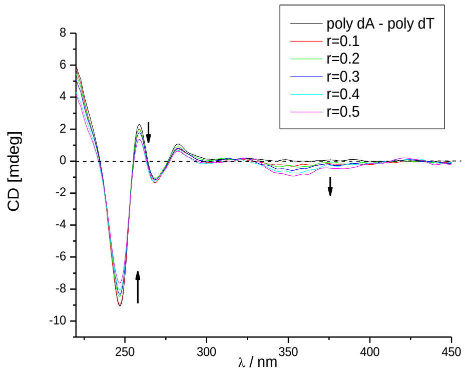

**AP5**	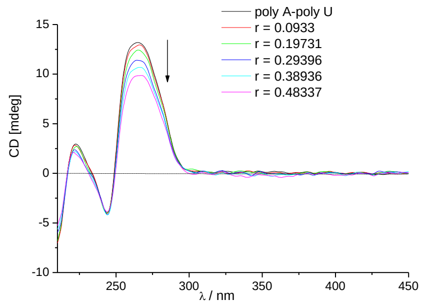	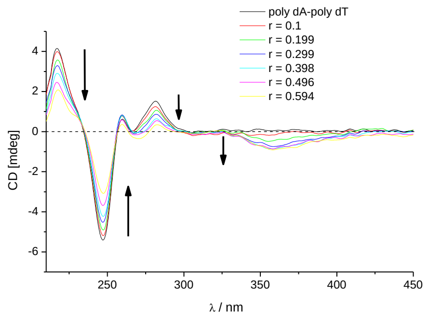
**AP6**	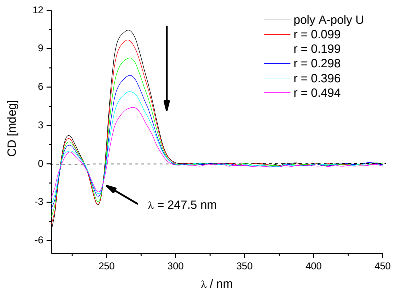	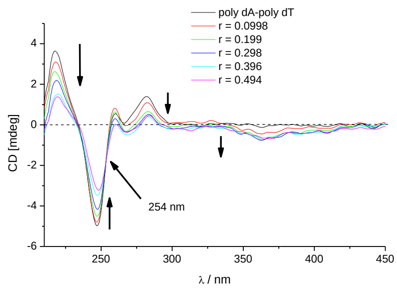

Figure S3. CD titration of poly A–poly U (c = 3.0 × 10^{-5} mol dm^{-3}) and poly dA–poly dT (c = 3.0 × 10^{-5} mol dm^{-3}) with **DP77, APH**, **AP3**, **AP3am, AP5** and **AP6** at molar ratios r = [compound] / [polynucleotide] (pH 7.0, buffer sodium cacodylate, I = 0.05 mol dm^{-3}).

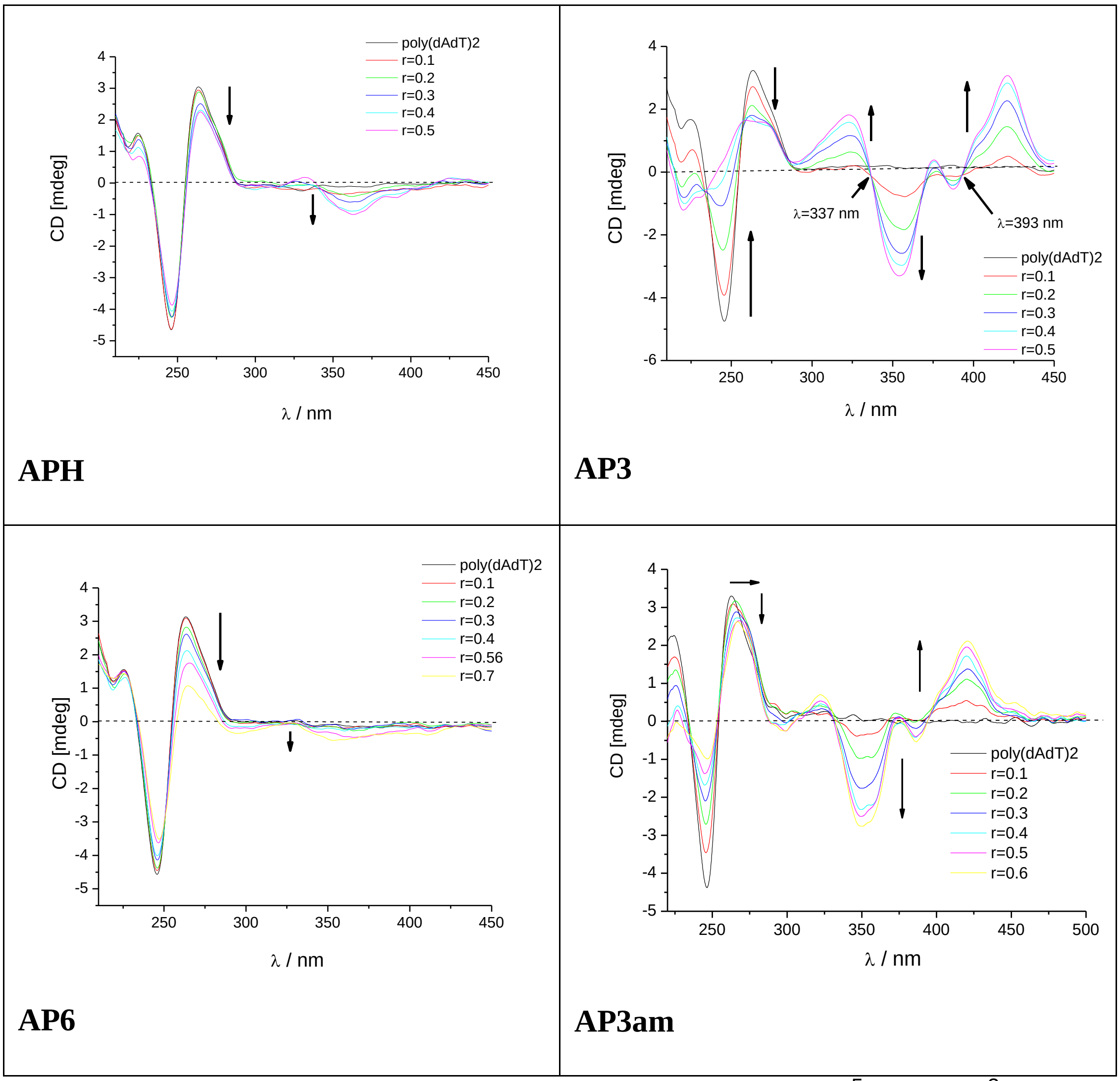

Figure S4. CD titration of poly dAdT–poly dAdT (c = 3.0 × 10^{-5} mol dm^{-3}) with **AP3**, **AP3am** and **AP6** at molar ratios r = [compound] / [polynucleotide] (pH 7.0, buffer sodium cacodylate, I = 0.05 mol dm^{-3}).

Table S1. Groove widths and depths for selected nucleic acid conformations [1].

	Groove width [Å]		Groove depth [Å]	
	major	minor	major	minor
[b] poly dAdT–poly dAdT	11.2	6.3	8.5	7.5
[c] poly dA–poly dT	11.4	3.3	7.5	7.9
[a] poly rA–poly rU	3.8	10.9	13.5	2.8
[b] poly dGdC–poly dGdC	13.5	9.5	10.0	7.2

[a] A-helical structure (e.g. A-DNA). [b] B-helical structure (e.g. B-DNA); [c] C-helical structure (e.g. C-DNA).

1 a) Saenger, W. *Principles of Nucleic Acid Structure*, Springer-Verlag: New York, 1983; b) Cantor, C. R.; Schimmel, P. R. *Biophysical Chemistry*, WH Freeman & Co: San Francisco, 1980.
